# Multiple hormone secretion may indicate worse prognosis in patients with ectopic Cushing’s syndrome

**DOI:** 10.1007/s42000-019-00163-z

**Published:** 2020-01-16

**Authors:** Ieva Lase, Ieva Strele, Malin Grönberg, Gordana Kozlovacki, Staffan Welin, Eva Tiensuu Janson

**Affiliations:** 1 Department of Medical Sciences, Endocrine Oncology Unit, Uppsala University, SE-751 85 Uppsala, Sweden; 2 Department of Public Health and Epidemiology, Riga Stradins University, Dzirciema street 16, Riga, LV-1007 Latvia

**Keywords:** Ectopic Cushing’s syndrome, ACTH, Neuroendocrine, Hypercortisolism, Multiple hormone secretion, Hypokalemia

## Abstract

**Purpose:**

Ectopic Cushing’s syndrome (ECS) caused by an ACTH secreting neuroendocrine neoplasm (NEN) is a rare and challenging condition. We aimed to detect predictive and prognostic parameters for ECS patients identified from a retrospective, comprehensive cohort of NENs treated at a tertiary referral center.

**Methods:**

Medical records of 886 patients with NENs were reviewed. We identified 51 patients with ECS (33 females/18 men); mean age 52 ± 15 years (SD). Clinical parameters including symptoms, biochemical markers, and survival were extracted and further analyzed.

**Results:**

The primary tumor was located in the thorax (*n* = 28) or pancreas (*n* = 15) or was of unknown primary origin (*n* = 8). In 30 patients, tumor and ECS were diagnosed simultaneously. In 12 patients, the NEN diagnosis preceded ECS development, with a median time of 43.5 months (range: 9–96), and 10 of these showed radiological tumor progression at ECS diagnosis. Twenty-one patients had multiple hormone secretion, which correlated with shorter overall survival (OS), *p* = 0.012 (HR 2.4 (95% CI 1.2–4.9)), as did high morning cortisol, *p* = 0.037 (HR 2.3 (1.0–5.2)), higher tumor grade, *p* = 0.044 (HR 2.3 (1.0–5.1)), and diabetes, *p* = 0.050 (HR 2.4 (1.0–6.0)).

**Conclusions:**

Multiple hormone secretion, high morning cortisol, higher tumor grade, and diabetes were correlated with shorter OS. Development of ECS in patients with a non-functioning NEN may indicate tumor progression. Multiple hormone secretion should be considered as a bad prognostic sign in ECS patients and should lead to intensified clinical management.

## Introduction

High levels of plasma cortisol may be due to several different medical disorders but they almost always lead to a severe health condition in the patient. Endogenous Cushing’s syndrome (CS) has an estimated incidence of 0.2–5.0 per million people per year [1]. In 5–10% of these, development of CS is due to an ectopic production of corticotrophin-releasing hormone (CRH) or ACTH by a non-pituitary tumor (ectopic ACTH-dependent CS) [1, 2].

The clinical picture of ectopic Cushing’s syndrome (ECS) can be the same as in Cushing’ s disease, but the rapid onset, severity, and progress of symptoms may suggest an ectopic source [2]. The clinical signs and symptoms include hypertension, high levels of blood glucose (often manifest diabetes mellitus), hypokalemia and metabolic alkalosis, and psychopathology and neurocognitive disorders, among many other manifestations. Severe infections, often caused by opportunistic agents, are also frequently seen due to the immunosuppressive action of cortisol.

The most well-known tumor responsible for ectopic ACTH production is small cell lung carcinoma (SCLC), and among SCLCs, 3.5–50% is reported to produce ACTH [2]. The reported prevalence range of ECS in patients with SCLC is very wide and probably depends on referral patterns of the patients to specialized clinics as well as small cohort sizes (selection bias) [2]. Most of these tumors lack immunohistochemical evidence of neuroendocrine differentiation and are highly malignant with extremely rapid clinical deterioration and short survival, which also can influence the reported prevalence. The second most common cause of ectopic ACTH production is neuroendocrine neoplasms (NENs) of various origins, such as lung 5–40%, pancreas 7.5–42%, and thymus 5–42% [2]. There are published case reports of ECS in connection with other types of malignancies as well, for example, pheochromocytoma [3, 4], medullary thyroid cancer [5–7], paraganglioma [8, 9], prostate cancer [10, 11], and even more rare ectopic sources of ACTH secretion [12–15]. Patients with genetically inherited syndromes, which are associated with a risk of development of NENs, can also develop ECS [16–19]. In 12.5–19% of patients with ECS, the source of ACTH secretion is never found [20, 21].

Because of the rarity of ECS, most of the publications are clinical case reports. In the case series published [20–34], the number of included patients is usually less than 50. Only two centers have published studies of > 100 patients with ECS [23, 35]. Thus, very little is known about the epidemiological, clinical, and molecular features of ACTH secreting NENs, and there is, to our knowledge, only one study published about predictive and prognostic markers of ECS in patients with NENs, indicating that severe hypercortisolism, hypokalemia, diabetes mellitus, and distant metastases were negative predictive factors [35]. The phenomenon of multiple hormone secretion has been described mainly in pancreatic NENs [36–41], while the impact of multiple hormone secretion in patients with ECS is yet unknown.

The aim of this study was to retrospectively analyze a cohort of NEN patients with ECS, referred to a tertiary NET center, in order to describe clinical symptoms and biomarkers of predictive and prognostic impact to identify parameters of clinical significance.

## Materials and methods

### Patients

At our tertiary referral center, we retrospectively reviewed the records of 886 patients with NENs (excluding small bowel NENs), referred to the Department of Endocrine Oncology, Uppsala University Hospital, between 1986 and 2014, to systematically search for patients with ECS. Patients with SCLC were not included in our cohort, since these tumors are not treated at our center and are hypothesized to have a different mechanism behind ACTH production from that of NENs. Fifty-one patients (6%) with ECS were identified. The pituitary/adrenal source of CS was excluded initially by hormonal analysis (serum and urinary cortisol, ACTH), functional tests (for example, low- and high-dose dexamethasone test, CRH stimulation test), imaging (CT and MR), and sinus petrosus sampling (IPSS).

### Data

Data were collected from medical records at Uppsala University Hospital or local hospitals where the first visit took place and included sex, age at diagnosis of tumor and/or ECS, approximate time from first symptoms of hypercortisolism to diagnosis of ECS, clinical symptoms, complications of ECS, blood pressure (BP), body mass index (BMI), and survival data. BP was divided into two groups, as normal (≤ 139/89 mmHg) or arterial hypertension (≥ 140/90 mmHg), according to the current guidelines for classification of BP [42]. Survival data were analyzed until March 2018.

All the collected clinical and biochemical parameters were retrospective; they were recorded at NENs and/or ECS diagnosis and before any symptomatic or tumor-directed treatment was started. The accuracy of the data on clinical symptoms and complications depends on how completely the medical history was collected and documented.

Biochemical data included potassium and glucose levels. Hypokalemia was defined as potassium levels < 3.5 mmol/l. Hormone levels recorded included ACTH (ng/l), urine free cortisol (nmol/24 h), morning and midnight serum cortisol (nmol/l), and other relevant hormones if the levels were abnormal.

Data regarding histological examination (when available) were extracted from medical records; no new immunohistochemical investigations were performed. For the grading of tumors of gastrointestinal origin (GEP-NENs), the WHO 2010 classification of tumors of the digestive system was used [43], and for the grading of thoracic NENs, the WHO 2014 classification of lung cancer was used [44]. In the case of repeated biopsies and histopathological examinations, the highest Ki67 was used in analyses.

### Evaluation of hormone levels

The study covered a long time period, and hormone levels were analyzed at different laboratories and with different methods. Therefore, the upper reference varied slightly between 40 and 72 ng/l for ACTH, between 230 and 372 nmol/24 h for urine free cortisol (UFC), and between 540 and 800 nmol/l for morning and 300–330 nmol/l for evening plasma cortisol. For the calculation of average hormone levels and for analysis of these data, those differences were not considered of importance, since almost all patients had very high hormone levels. Multiples of the upper limit of normal (UNL) were used as well to describe hormone levels in the cohort. When analyzing survival, levels of ACTH, UFC, and cortisol were divided into two groups (low vs. high) by using the median hormone level as cut-off.

### Statistical analysis

Patient characteristics and biochemical findings were summarized by descriptive statistics: normally distributed data were described as means ± standard deviation and data with skewed distribution and/or outliers were described as medians, accompanied by the 25th–75th percentile ranges (Q1-Q3). The defined event was death from any cause. Overall survival (OS) was defined as time from diagnosis of NEN and/or ECS until date of death, or if event was not found, censored at date of last observation, 1st of March 2018. Kaplan-Meier plots were used for survival analysis, and the log-rank test was used for comparison. Cox proportional regression was performed for the estimation of hazard ratios (HRs) and confidence intervals (CIs). Correlation was estimated using Spearman’s correlation test. The level of statistical significance was set at 0.05. All statistical analyses were performed using IBM SPSS Statistics software (v24, USA).

## Results

As the data were collected retrospectively from patient records, not all the preferred parameters were available for all patients.

### Patients

Among the 51 cases of ECS, there were 18 males and 33 females. Mean age at tumor diagnosis was 51 ± 15 years (SD) and at diagnosis of ECS 52 ± 15 years (SD). Clinicopathological characteristics of the cohort are presented in Table 1. The primary tumor was located in the pancreas in 15 patients and in the thorax (bronchial or thymus NEN) in 28 patients. There were no patients with ECS due to medullary thyroid carcinoma identified in our cohort. A majority of the pancreatic tumors were G2 and most of the thoracic tumors were atypical carcinoids (patients with SCLC not included). Among the eight patients with ECS and NENs of unknown primary origin, three patients had liver metastases at diagnosis, but the primary tumor was never found, while in five patients, a tumor could not be found despite extensive work-up, including octreotide scintigraphy and/or positron emission tomography. Thirty-four patients had documented metastatic disease to one (*n* = 15) or more (*n* = 19) locations at the time of ECS diagnosis.

**Table 1:** Clinicopathological characteristics of 51 patients with ectopic Cushing’s syndrome

Variable	*N* (%)	Symptoms	*N* (%)
Gender		Hypertension	36 (71)
Female	33 (65)	Diabetes	36 (71)
Male	18 (35)	Moon face	35 (69)
Age at diagnosis of tumor (years)		Central obesity	32 (63)
< 40	13 (25)	Muscle weakness	24 (47)
41–70	28 (55)	Ankle edema	21 (41)
> 71	5 (10)	Bruising	19 (37)
No tumor found	5 (10)	Psychiatric symptoms	16 (31)
Tumor localization		Hirsutism	14 (27)
Pancreas	15 (29)	Thin skin	8 (16)
Bronchial NENs	24 (47)	Striae	7 (14)
Thymus	4 (8)	Hyperpigmentation	4 (8)
Unknown	8 (16)	Amenorrhea	2 (4)
Metastasis localization at diagnosis of tumor		Acne	1 (2)
Liver	23 (45)	Hyperhidrosis	1 (2)
Lymph nodes	12 (23)	Baldness	1 (2)
Bone	4 (8)	No typical Cushing symptoms	1 (2)
Lung	4 (8)	Complications	
Other	4 (8)	Bacterial infections/sepsis	25 (49)
No metastasis	20 (39)	Thromboembolic complications	11 (22)
Tumor grade		Fungal infections	8 (16)
GI-NENs (pancreatic)		Osteoporosis/osteopenia/fractures	8 (16)
G1Ki67 < =2%	0 (0)		
G2Ki67 3–20%	9 (60)		
G3Ki67 > 20%	2 (13)		
No data	4 (27)		
Lung NENs (bronchial + thymic)			
Typical	1 (4)		
Atypical	14 (50)		
NEC (excluding SCLC)	8 (28)		
No data	5 (18)		

*GI-NEN* gastrointestinal neuroendocrine neoplasia, *NEN* neuroendocrine neoplasia, *NEC* neuroendocrine carcinoma, *SCLC* small cell lung carcinoma

Immunohistochemical staining for ACTH/CRH was performed in 24 patients: 15 patients were positive for ACTH, two for CRH, and one for both hormones. Six patients had negative staining for both ACTH and CRH (in five of the patients, the immunohistochemical staining was performed on liver metastases and in one patient on the primary tumor) despite metastatic NEN, high circulating ACTH levels and no radiological evidence of pituitary disease. Two of these latter patients had a very acute debut of disease with extremely high cortisol/ACTH levels and need for intensive care due to hypokalemia.

Thirty patients (59%) were diagnosed simultaneously with ECS and tumor, while 12 (23%) had an initial diagnosis of a NEN without ECS, based on absence of clinical symptoms of CS, normal potassium, and ACTH/cortisol levels. Among those 12, there was a wide difference in time from diagnosis of tumor to development of ECS, with median time 43.5 months (range: 9–96). Ten of those 12 patients had radiological tumor progression at the time of diagnosis of ECS.

Nine patients (18%) were initially diagnosed with ECS without a detectable tumor. In five of these patients, ECS was defined as occult since neither primary tumor nor metastatic disease was found and pituitary origin was excluded. One of those patients had cyclic Cushing’s syndrome with a need for intensive care during periods of excessive cortisol secretion. In the whole cohort, the median time from first Cushing symptoms to diagnosis of ECS was 2 months (range: 0–12).

A summary of the symptoms and most frequent complications of hypercortisolism observed in the cohort is presented in Table 1. Fifty patients had at least one clinical sign related to Cushing’s syndrome at diagnosis.

Diabetes mellitus and arterial hypertension at diagnosis was found in 36 (71%) of patients, respectively. BP at diagnosis was unknown for eight patients.

### Biochemical findings

Patients with ECS had a tendency towards hyperglycemia and hypokalemia. Information about the potassium level at diagnosis was available for 44 patients, and 33 (75%) of those had hypokalemia. Thirty-two percent of patients had a potassium level < 2.4 mmol/l, 23% had levels between 2.5 and 2.9 mmol/l, and 20% had levels between 3 and 3.4 mmol/l at diagnosis.

Almost all patients had very high hormone levels. ACTH was 1–2 × ULN in 16 patients, 3–10 × ULN in 21 patients, and > 11 × ULN in six patients. UFC was 1–2 × ULN in three patients, 3–20 × ULN in 26 patients, 21–99 × ULN in seven patients, and in eight patients more than 100 × ULN. Mean values of biochemical data and median hormone levels are summarized in Table 2.

**Table 2:** Biochemical data and hormone levels for patients with ectopic Cushing’s syndrome

Laboratory findings	Number	Mean value ± SD	Min-max
Potassium (mmol/l)	44	2.9 ± 0.7	1.9–4.4
Glucose (mmol/l)	40	10.5 ± 6.2	3.6–29
Laboratory findings	*N*	Median	25th–75th percentile
ACTH (ng/l)	47	156	92–341
UFC (nmol/24 h)	42	4247	2726–27,117
Serum morning cortisol (nmol/l)	40	1318	822–2206
Serum midnight cortisol (nmol/l)	17	1184	724–1823

Upper reference for ACTH 40–72 ng/l, for 24 h for urine free cortisol 230–372 nmol, for morning plasma cortisol 540–800 nmol/l and for evening plasma cortisol 300–330 nmol/l

*ACTH* adrenocorticotropic hormone, *UFC* urine free cortisol

Twenty-one patients (41%) (10 pancreatic NENs and 11 thoracic NENs) had concomitant hypersecretion of other hormones than ACTH from their tumor (Table 3). Five of those had simultaneous hypersecretion of two or more hormones in addition to ACTH. 5-HIAA was elevated in 11 patients, with a median level of 461 ng/ml (range: 134–2151): normal range < 50 ng/ml; eight of these had thoracic NENs, two pancreatic NENs, and one unknown primary tumor. In 17 patients, hypersecretion of ACTH and other hormones was discovered simultaneously, while two patients developed ECS first, followed by hypersecretion of other hormones some years later. Two patients had hypersecretion of hormones, resulting in clinical syndromes with typical clinical symptoms (insulinoma and gastrinoma) for 36 and 149 months, respectively, prior to ECS.

**Table 3:** Overview of concomitant hypersecretion of other hormones than ACTH only and relation to tumor site in patients with ectopic Cushing’s syndrome

Hormone secretion in addition to ACTH	Patients with multiple hormone secretion *N* = 21	Tumor site			
		Pancreas *N* = 9	Lungs *N* = 10	Thymus *N* = 1	Unknown *N* = 1
5-HIAA	8	–	6	1	1
Calcitonin	3	–	3	–	–
Gastrin	2	2	–	–	–
Glucagon	3	3	–	–	–
Calcitonin+5-HIAA	2	1	1	–	–
Gastrin+5-HIAA	1	1	–	–	–
Gastrin + Proinsulin	1	1	–	–	–
Insulin + Gastrin + VIP	1	1	–	–	–

*ACTH* adrenocorticotropic hormone, *VIP* vasoactive intestinal peptide, *5-HIAA* 5-hydroxyindoleacetic acid

Four patients had primary hyperparathyroidism with elevated levels of PTH and persistent hypercalcemia (albumin corrected or ionized calcium). All these patients had thoracic NENs (three atypical bronchial carcinoids and one thymus carcinoid). They were diagnosed with ECS in the 1990s when possibilities for genetic testing for MEN1 were limited, but two of them showed a clinical picture of MEN1 with hyperparathyroidism, pituitary adenoma, and NEN.

Potassium levels were negatively correlated to blood glucose (*ρ* = − 0.5, *p* = 0.002), ACTH (*ρ*= − 0.4, *p* = 0.01), UFC (*ρ*= − 0.5, p = 0.002), morning cortisol (*ρ* = − 0.6, *p* < 0.001), and evening cortisol (*ρ* = − 0.5, *p* = 0.032) levels. Blood glucose was positively correlated to morning cortisol (ρ = 0.5, *p* = 0.004). BP and Ki67 did not correlate with potassium or hormone levels.

### ECS and survival

At the end of the study period, ten patients were still alive and 36 had died. Data were not available for five patients. Although the concrete cause of death in ECS patients may be difficult to define due to a very complex clinical picture and overlap of comorbidities, the following causes were retrieved from medical records: progress of tumor (*n* = 18), infections and other ECS-related comorbidities (*n* = 8), complications after adrenalectomy (*n* = 3), multi-organ failure (*n* = 1), other malignancies (*n* = 1), and myocardial infarction (*n* = 1). Cause of death was unknown in four patients.

Average follow-up time from tumor diagnosis until end of the study period was 46 months (range 12–104) and from the time of ECS diagnosis to the end of the study period 33 months (range 6–83). Median OS from tumor diagnosis was 46 months (95% CI 20–71), with a 5-year survival of 41%. OS from the time of ECS diagnosis was 33 months (95% CI 16–50), with a 5-year survival of 30%. There was no difference in OS between thoracic NENs and pancreatic NENs.

**Fig. 1:**
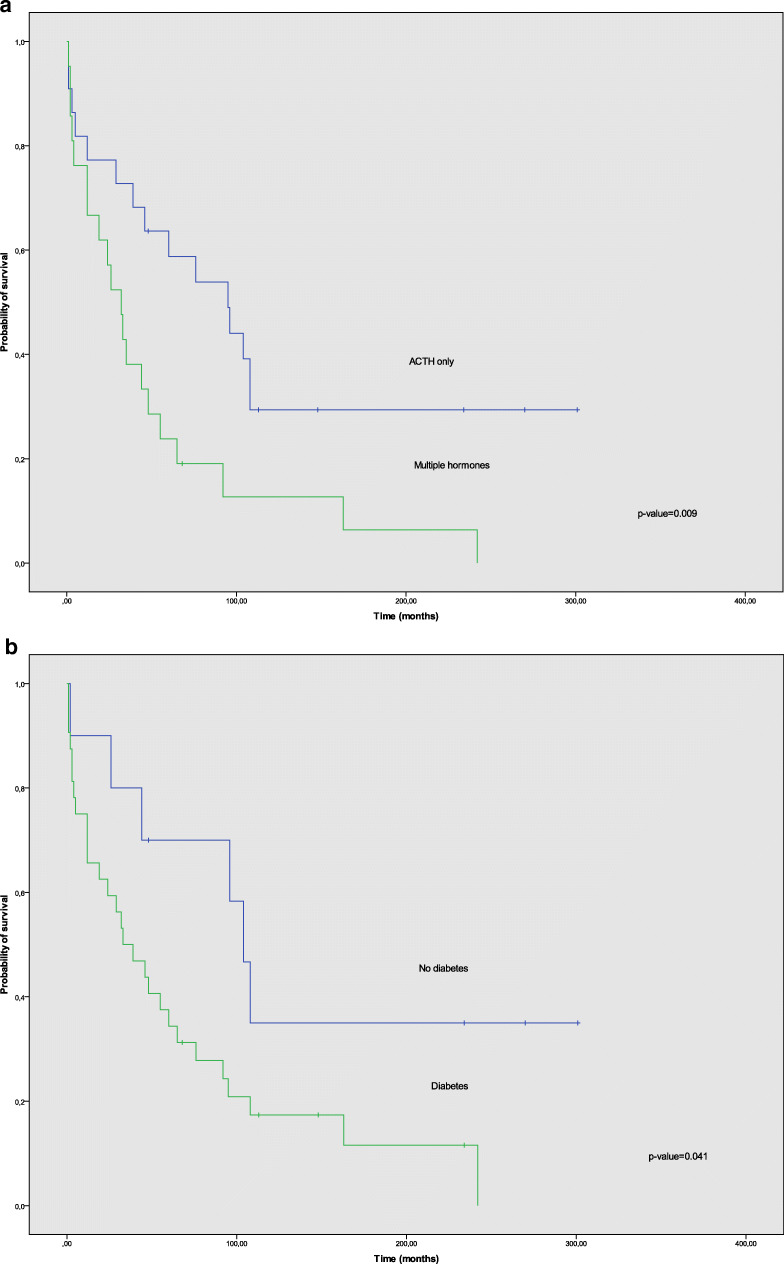

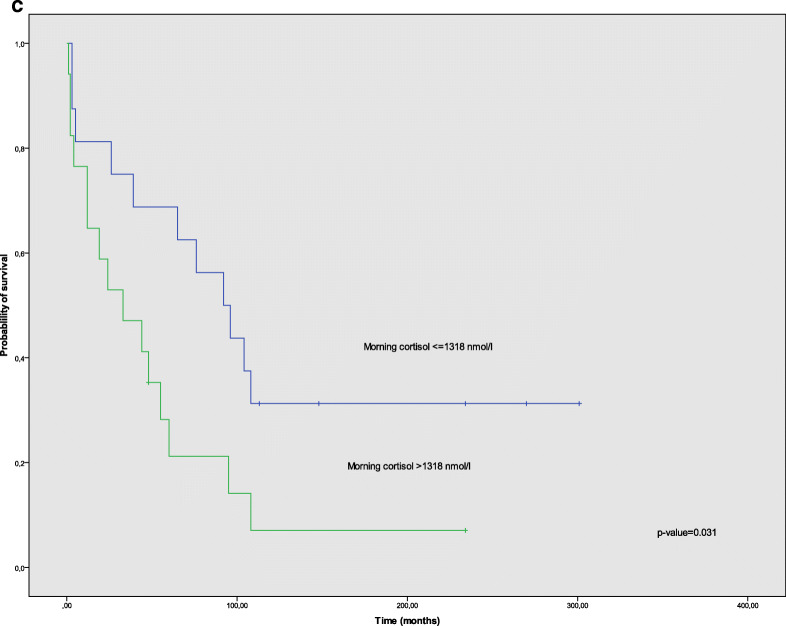
Kaplan-Meier overall survival (OS) curves for patients with ectopic Cushing’s syndrome (ECS) according to different clinical and biochemical parameters. **a** OS according to either tumor secretion of only adrenocorticotropic hormone (ACTH) or ACTH + other hormones (multiple hormone secretion). **b** OS according to presence of diabetes at diagnosis of tumor. **c** OS according to morning cortisol levels at diagnosis

Patients who died due to ECS-related comorbidities had a shorter survival, 5 months vs. 18 months, compared to those who died due to tumor progression (*p* = 0.001). Patients with multiple hormone secretion had shorter survival, 32 months vs. 95 months, compared to those with ECS only (*p* = 0.009), confirmed by univariate Cox regression analysis, *p* = 0.012 (HR 2.4 (95% CI 1.2–4.9)). Multiple hormone secretion was a statistically significant independent variable in multivariate Cox regression analysis, *p* = 0.044 (HR 3.9 (95% CI 1.04–14.7)). Furthermore, shorter OS was found in patients with diabetes: 33 months vs. 104 months (*p* = 0.041), higher than median morning cortisol levels: 33 months vs. 92 months (*p* = 0.031), and tumors with Ki67 > 20%: 19 months vs. 65 months (*p* = 0.036), compared to non-diabetic patients, patients with lower than median morning cortisol, and tumors with Ki67 < 20%, respectively. Furthermore, patients with hypokalemia at diagnosis showed a trend towards shorter OS in our cohort: 32 vs. 76 months (*p* = 0.067). Survival data obtained from Cox regression models are summarized in Table 4 and Kaplan-Meier OS curves for those parameters are shown in Fig. 1.

**Table 4:** Univariate and multivariate analysis of prognostic parameters of survival in ectopic Cushing’s syndrome

Variable		HR (95% CI)	*p* value
Univariate analysis			
Hormone secretion	ACTH only vs. multiple hormones	2.4 (1.2–4.9)	0.012
Morning cortisol nmol/l	≤1318 vs. > 1318	2.3 (1.0–5.2)	0.037
Tumor grade (WHO 2010)	I + II vs. III	2.3 (1.0–5.1)	0.044
Diabetes	Yes vs. No	2.4 (1.0–6.0)	0.050
Potassium mmol/l	< 3.5 vs. ≥ 3.5	2.2 (0.9–5.5)	0.077
Evening cortisol nmol/l	≤ 1184 vs. >1184	2.5 (0.7–9.2)	0.164
Tumor localization	Thoracic vs. pancreas	0.7 (0.3–1.5)	0.432
ACTH ng/ml	≤ 156 vs. > 156	1.2 (0.6–2.5)	0.530
UFC nmol/24 h	≤ 4247 vs. > 4247	0.9 (0.4–1.8)	0.744
Multivariate analysis			
Hormone secretion^a^	ACTH only vs. multiple hormones	3.9 (1.0–14.7)	0.044

Hazard ratio (HR) and 95% confidence intervals (CIs) obtained from Cox regression models. ACTH, UFCm, and cortisol are divided in two groups (low vs. high) by using the median hormone level as cut-off

*ACTH* adrenocortical hormone, *UFC* urine free cortisol

^a^Adjusted for morning cortisol levels, diabetes, Ki67, and hypokalemia

## Discussion

In this comprehensive cohort of patients with ACTH-producing NENs, we identified multiple hormone secretion as the strongest indicator of worse prognosis. In addition, high morning cortisol, high blood glucose (diabetes mellitus), and higher tumor grade had a similar impact on OS, confirming results from a previous study [35]. Hypokalemia showed a tendency towards shorter OS as well.

ACTH production from NENs is a very rare but serious condition, almost always creating a big challenge for physicians as regards diagnostic and treatment issues. In this comprehensive cohort from Uppsala ENETs Centre of Excellence, 6% of all patients with NENs were identified as having ECS in contrast to another similar cohort where only 3.2% were identified [32]. There was a predominance of women (ratio 2:1) in the present material, which is consistent with data in other series [23, 28, 32]. Mean age at diagnose of ECS was 52 years in contrast to a number of previous studies, where it varied from 27.6 to 49 years [20, 21, 23, 24, 26, 32]. The age differences between different cohorts could be explained by the proportion of different NEN types included, since thymus NENs tend to occur in younger persons [29, 34], while SCLC is more frequent in older individuals [45].

SCLC patients were not included in our cohort. SCLC with ECS has a very serious course, with rapid deterioration and short survival. Had those patients been included, it would have been difficult to delineate the clinical course of ECS in other NENs, which was the purpose of our study. The different biology between SCLC and NENs is further indicated by the fact that the molecular defects behind ectopic ACTH secretion may not be similar [1]. Molecular biology studies have proposed different mechanisms for ACTH production between poorly differentiated tumors, SCLC, and neuroendocrine tumors. For example, SCLC cells process proopiomelanocortin (POMC) in an aberrant way, which results in the release of ACTH precursors of large molecular weight, such as pro-ACTH, while NET cells process POMC, normally releasing large amounts of intact ACTH into the circulation [46].

ECS has been reported to occur more frequently in thoracic NENs than in pancreatic NENs [2, 47], which is supported by the results of the present study (55 vs. 29%). However, the incidence of ECS due to pancreatic NENs was higher in our cohort than previously reported [20, 23]. This is probably due to a selection bias, since our study was performed in a specialized center for endocrine oncology. We could not detect any difference in OS based on origin of the primary tumor.

In eight patients in our cohort, the primary tumor was never found. In these cases, ECS was suspected either due to metastatic disease (*n* = 3) or to negative pituitary MRI/IPSS in patients with a very aggressive course of Cushing’s syndrome and absence of detectable tumor (*n* = 5). It should also be noted that the study period covered almost 30 years, and some patients were investigated before the era of positron emission tomography, which could explain why some small primary tumors were never found.

More than a half of the patients were diagnosed simultaneously with tumor and ECS. In patients in whom ECS developed subsequently, the ECS diagnosis was almost always associated with radiological progression of disease. Median OS in this cohort was 46 months, which is longer than previously reported [28, 32]. This observed difference may depend on differences in patients included, since we did not include SCLC.

There is a study showing significantly shorter 5-year survival in ECS patients compared to those with non-functioning NENs [32]. Mortality is high during the first time period after ECS diagnosis, mostly due to complications of hypercortisolism [41]. We found very high hormone levels at ECS diagnosis, suggesting that even though a patient with ECS has a histopathologically well-differentiated tumor (most of our patients had G2 tumors), prognosis may be worse than in patients with non-functioning NENs. Others have also described hypercortisolism as a negative predictive factor in ECS [35].

Almost half of our patients had multiple hormone secretion, which usually appeared simultaneously with the ECS diagnosis. The hormone which was elevated most frequently was U-5-HIAA, dominating in thoracic NENs, followed by calcitonin. There was no evidence of concurrent presence of small bowel NEN or medullary thyroid carcinoma. Patients with multiple hormone secretion had significantly shorter OS. Multiple hormone secretion has been described in pancreatic NENs [36–41], suggesting that there is a trend towards shorter survival in patients with multiple hormone secretion [36, 40]. However, there are as yet no published data on how concomitant hormone secretion influences OS in thoracic NENs or in patients with ECS. Our findings suggest that multiple hormone secretion can also be found in thoracic NENs, leading to worse prognosis compared to that of patients with ECS only. This indicates that at diagnosis of ECS, other hormones should be measured, especially U-5-HIAA and calcitonin, in order to identify patients who need intensified treatment and follow-up.

Severe hypokalemia, often requiring long periods of hospitalization and intensive care, is a well-known clinical challenge in patients with ECS. This is fully supported by the results of the present study, where 75% of patients had potassium levels < 3.5 mmol/l at diagnosis. Hypokalemia was inversely related to hormone levels and negatively affected OS compared to normokalemic patients in our cohort (32 vs. 76 months), which confirms the significance of this metabolic disturbance. Presence of diabetes at diagnosis also had a negative impact on OS compared to non-diabetic ECS patients (33 vs. 104 months). Davi et al. describe similar findings in their cohort regarding negative prognostic factors in ECS [35].

In conclusion, multiple hormone secretion can occur in ECS patients with NENs of both thoracic and pancreatic origin. Patients with multiple hormone secretion as well as high morning cortisol levels and diabetes have worse prognosis, and these patients should be monitored carefully and probably treated more aggressively. Development of ECS in patients with previously known non-functioning NEN may indicate radiological progression of disease. To further improve our knowledge about ECS, multicenter studies are needed with large, prospective cohorts and standardized inclusion criteria.
